# Endoscopic drainage and one-stage titanium clip closure for appendiceal root abscess mimicking cecal submucosal tumor: a case report

**DOI:** 10.3389/fmed.2026.1915944

**Published:** 2026-08-27

**Authors:** Yanping Dai, Yang Liu, Jun Ye

**Affiliations:** 1 Department of Gastroenterology, Chonggang General Hospital, Chongqing, China; 2 Department of Gastroenterology, The First Affiliated Hospital (Southwest Hospital) of Army Medical University (Third Military Medical University), Chongqing, China

**Keywords:** case report, endoscopic incision and drainage, endoscopic ultrasonography, periappendiceal abscess, submucosal mass

## Abstract

Periappendiceal abscess stands as a frequent complication stemming from acute appendicitis. One rare variant presents as a submucosal mass at the root of the appendix and cecum. Clinicians often misdiagnose this form at alarmingly high rates. Conventional management brings substantial surgical trauma. It also prolongs patients’ recovery periods. Whether minimally invasive endoscopic intervention works well for this distinct abscess subtype still lacks solid clinical proof. Whether minimally invasive endoscopic intervention works well for this distinct abscess subtype still lacks solid clinical proof. This report documents a single case of a 34-year-old female patient. Dull epigastric pain served as her chief complaint. Initial colonoscopy spotted a submucosal lesion around the appendiceal orifice. Contrast-enhanced abdominal CT and endoscopic ultrasonography (EUS) followed afterward. Combined with the golden diagnostic evidence of purulent fluid extruded by gentle intraoperative compression on the lesion, a definitive diagnosis of localized periappendiceal abscess confined to the appendiceal base was confirmed. Endoscopic incision and drainage were performed for lesion decompression. A tiny perforation measuring 3 mm emerged mid-procedure. Clinicians sealed the defect primarily with titanium clips. Abdominal paracentesis was applied concurrently to evacuate intraperitoneal gas. The patient recovered uneventfully after the operation. This atypical periappendiceal abscess easily mimics submucosal neoplasms, which raises diagnostic confusion. Endoscopic ultrasonography acts as the pivotal tool to achieve accurate differentiation. Endoscopic minimally invasive drainage delivers reliable safety and therapeutic efficacy. Intraoperative micro-perforations can be closed in one session via endoscopy. This strategy eliminates the need for open surgery. Its prominent minimally invasive merits make the technique worthy of broader clinical adoption.

## Introduction

Periappendiceal abscess develops as a common complication when acute appendicitis fails to receive timely intervention. Most patients report right lower quadrant pain, fever and palpable abdominal masses as core manifestations (1–3). These classic signs enable straightforward clinical judgment in routine practice. A distinct clinical scenario exists nonetheless. If pus collection localizes at the appendiceal base and bulges inward into the cecal lumen, submucosal mass will become its sole endoscopic sign. Typical appendicitis symptoms vanish in such cases. Clinicians frequently mistake this lesion for submucosal neoplasms, ranging from neuroendocrine tumors and gastrointestinal stromal tumors to leiomyomas (4–6). Misdiagnosis often triggers unnecessary endoscopic submucosal dissection (ESD) or radical surgical resection. Both interventions expose patients to extra trauma and elevated medical risks.

Three mainstream strategies are currently applied to manage periappendiceal abscess: conservative antibiotic therapy, percutaneous puncture drainage and surgical appendectomy (7, 8). All three approaches carry notable drawbacks. They inflict severe tissue injury, delay postoperative rehabilitation and raise the likelihood of adverse events. Endoscopic retrograde appendicitis therapy (ERAT) has been validated as a safe and effective modality for uncomplicated acute appendicitis and appendicolith removal (9–11). Few clinical reports, however, have addressed endoluminal incision and drainage paired with concurrent perforation repair for abscess lesions masquerading as submucosal masses. Prior publications omit critical operational details. Standardized abdominal puncture workflows, complete baseline lab values and tiered antibiotic protocols rarely appear in comparable case records. Few articles explain why drainage wounds demand immediate clip closure or clearly define which patients qualify for endoscopic abscess treatment.

A female patient was hospitalized with dull upper abdominal discomfort, while classic right lower quadrant tenderness was absent on physical examination. A submucosal lesion was identified on colonoscopy, and ESD was initially planned, yet characteristic abscess signs were detected via preoperative EUS, making endoscopic drainage replace the original scheme. Minor intraoperative perforation occurred and was closed uneventfully by endoscopy, and complete recovery was achieved through this ultra-minimally invasive management.

This case report intends to summarize diagnostic pitfalls linked to this atypical subtype of periappendiceal abscess. It also elaborates standardized endoscopic diagnostic and therapeutic workflows, alongside actionable management tactics for procedural perforations. Relevant insights herein may serve as practical references for frontline gastroenterologists.

## Case presentation

**Figure 1:**
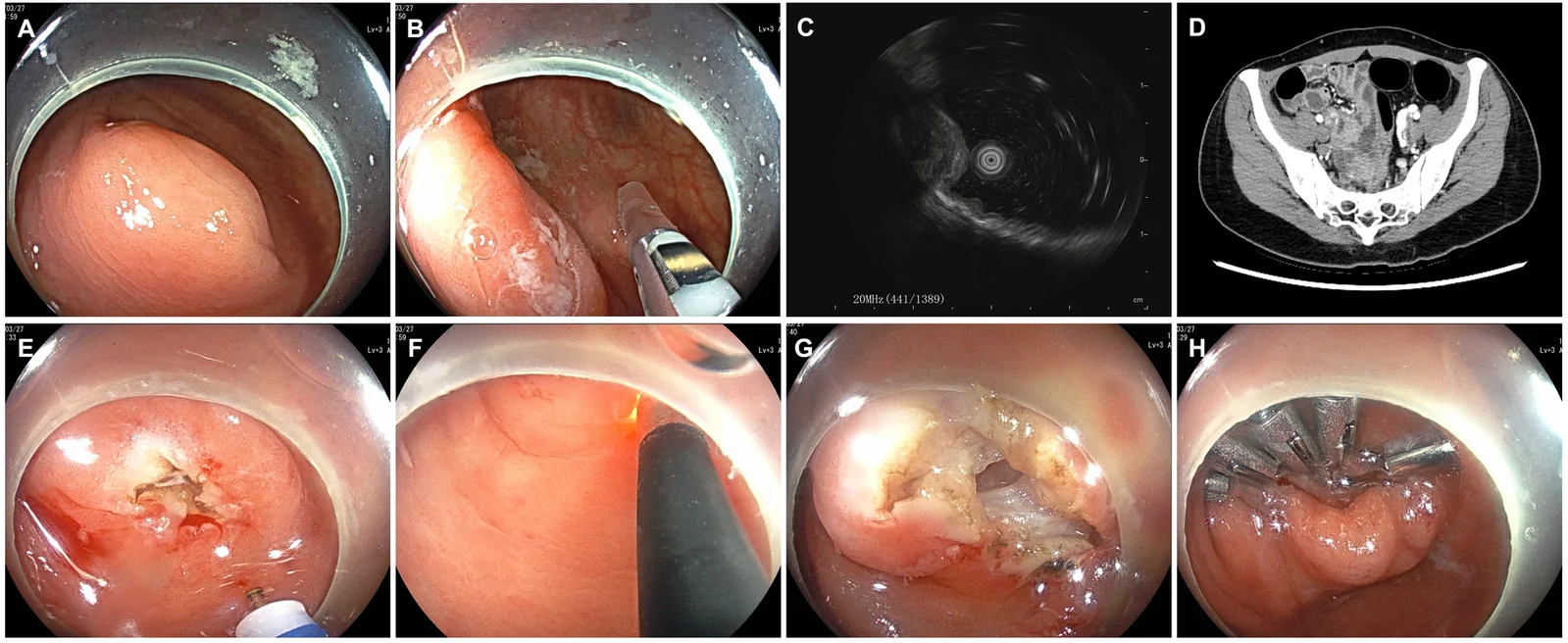
Imaging features and endoscopic treatment of periappendiceal root abscess. **(A)** Colonoscopy reveals a submucosal protuberance beside the appendiceal opening. **(B and C)** Endoscopic ultrasonography visualizes an extramural lesion originating outside the fourth intestinal wall layer with heterogeneous internal echoes and localized hypoechoic areas; fluid discharge can be seen after compression on the lesion. **(D)** Contrast-enhanced CT displays a well-demarcated hypodense lesion at the appendiceal base. **(E)** An IT knife is applied to make a central cut on the protruding lesion when the original appendiceal ostium cannot be recognized. **(F)** Purulent fluid drains out, subsequent saline lavage is conducted, and the cavity condition is observed via microendoscopy. **(G)** A 3-mm microperforation was detected intraoperatively on the intact bowel wall near the appendiceal opening. **(H)** An inflammatory mucosal break is found during the operation, and this break as well as the surgical incision are closed with titanium clips.

A 34-year-old woman received hospital admission on March 25, 2025, with a one-week history of persistent dull epigastric pain. No prior records of appendicitis, abdominal surgery, abdominal trauma or chronic systemic illnesses were documented in her medical history. Unprovoked dull epigastric discomfort emerged one week before hospitalization and lingered without remission. Fever, nausea, vomiting and diarrhea were all absent; gastric and anti-inflammatory drugs taken by herself failed to ease her discomfort. Normal results were obtained from abdominal ultrasonography and gastroscopy performed at another medical facility. A submucosal lesion adjacent to the appendiceal orifice was visualized under colonoscopy, which was tentatively diagnosed as a submucosal neoplasm (Figure 1A). Endoscopic resection was thus recommended, and she was transferred to our hospital for planned ESD. Admission physical examination revealed mild tenderness at the right periumbilical abdomen, without muscular tension, rebound tenderness or palpable masses, and bowel sounds were normal. Full baseline laboratory testing was completed upon admission, and all blood and inflammatory results are compiled within Table 1. Elevated white blood cells, neutrophil counts, CRP and procalcitonin confirmed localized infection without severe systemic sepsis. Liver, renal and electrolyte panels fell fully within normal limits. Imaging results: contrast-enhanced abdominal CT identified a quasi-circular hypodense lesion approximately 10 mm in diameter at the appendiceal root of the cecum in the right lower quadrant with clear margins, suggestive of a benign space-occupying lesion (Figure 1D). Before the scheduled ESD, endoscopic ultrasonography was arranged. Imaging manifested that the lesion originated outside the fourth layer of intestinal wall with mixed echo signals and local anechoic areas, accompanied by thickening of adjacent bowel wall, with a cross-section size of 10.6 mm × 8.3 mm. Gentle compression on the protuberance extruded white pus from its central bulge, establishing the diagnosis of periappendiceal abscess at the appendiceal root (Figures 1B,C).

**Table 1:** Comparison of blood routine and inflammatory index results of patients before and after treatment.

Classification	Detection indicators	Before treatment	After treatment	Normal reference range
Blood routine	White blood cell	11.50	7.46	(3.5∼9.5) × 10^9^/L
	Neutrophil	10.07	5.14	(1.8∼6.3) × 10^9^/L
	Neutrophil percentage	87.60	68.9	40%∼75%
	Lymphocyte	0.81	1.73	(1.1∼3.2) × 10^9^/L
	Monocyte	0.60	0.35	(0.1∼0.6) × 10^9^/L
	Eosinophil	0.01	0.23	(0.02∼0.52) × 10^9^/L
	Basophil	0.01	0.01	(0∼0.06) × 10^9^/L
Inflammatory indicators	C-reactive protein	40.76	1.59	0∼10 mg/L
	Procalcitonin	2.44	<0.03	0∼0.076 ng/mL

Once a confirmed diagnosis was reached, the original ESD plan was abandoned without delay, and endoscopic incision combined with drainage was adopted for subsequent intervention. The natural appendiceal orifice could not be located during repeated colonoscopic inspections. A tiny cut was made at the central region of the protuberance using an IT knife (Figure 1E), from which flocculent white pus spontaneously drained. The abscess pocket was lavaged repeatedly with normal saline until clear fluid flowed out. Further deep dissection was advanced toward the appendiceal base, and a miniature biliary endoscope was delivered to verify thorough evacuation of residual purulent contents (Figure 1F). A roughly 3-mm tiny perforation was discovered on intact intestinal mucosa adjacent to the appendiceal opening intraoperatively (Figure 1G), a complication stemming from the friable intestinal wall altered by severe inflammation. The perforation site together with the drainage wound was sealed tightly at once with titanium clips (Figure 1H). A standardized abdominal paracentesis was then performed. We disinfected the right lower quadrant skin and placed sterile drapes before inserting an 18-gauge puncture needle vertically into the peritoneal cavity under endoscopic visual guidance. Only trapped intraperitoneal gas escaped during aspiration; no ascites or purulent fluid was retrieved. This step lowered intra-abdominal pressure and prevented distension-related toxic symptoms triggered by intraoperative air leakage.

Postoperative care measures included fasting, intravenous fluid support, and combined anti-infection therapy with ceftizoxime and ornidazole. No prophylactic antibiotics were administered before endoscopic intervention. Intravenous ceftizoxime sodium 2 g three times daily combined with ornidazole 1 g once daily was prescribed for five consecutive postoperative days, with dosage adjustments guided by daily inflammatory marker trends. On postoperative day 1, the patient only presented mild distending pain in the right lower abdomen without fever. All abdominal pain vanished completely on postoperative day 2, with restoration of flatus and defecation, and oral diet was then resumed. The patient was discharged after follow-up inflammatory markers returned to normal levels, and she was highly satisfied that no visible surgical scars were left on her abdomen.

Follow-up observation: Telephone interview and imaging reexamination were conducted 2 months after operation. The patient had no recurrent abdominal pain, fever, diarrhea, hematochezia or other discomforts. Abdominal CT scan displayed complete regression of the abscess at the appendiceal root with well-recovered local tissue structure (Supplementary Figure S1).

## Discussion

Periappendiceal abscess manifesting as submucosal protuberance at the appendiceal root represents an atypical clinical variant. It is highly prone to misdiagnosis as submucosal neoplasms and poses a persistent diagnostic challenge in routine practice. Conventional colonoscopy only visualizes superficial mucosal contours and fails to characterize deep-seated lesions (12). In contrast, endoscopic ultrasonography delineates the originating layer, internal echogenicity and anechoic fluid zones with sharp resolution (13). When combined with pus extrusion upon intraoperative compression, EUS enables rapid confirmation of abscess, greatly cutting down misdiagnosis risks. It serves as an irreplaceable preoperative modality for differential diagnosis of such atypical lesions.

Endoscopic incision and drainage constitutes a vital minimally invasive therapy for periappendiceal abscess. Timely prevention and standardized management of intraoperative perforations determine whether minimally invasive intervention succeeds without conversion to open surgery. A tiny 3 mm perforation emerged during this case, whose underlying mechanism is highly representative. Severe inflammatory edema at the appendiceal root renders the bowel wall extremely fragile. The inherently thin cecal wall is susceptible to laceration from incision, dissection and lavage maneuvers. Blurred appendiceal orifice and distorted local anatomy further raise procedural hazards. These perforations are minor, localized iatrogenic injuries with mild tissue contamination and no extensive necrosis. They differ fundamentally from perforations induced by tumors or corrosive lesions, creating favorable conditions for primary endoscopic closure.

Immediate titanium clip closure of drainage defects carries clear pathophysiological justification. Unclosed mucosal gaps create a direct channel between the abscess cavity and peritoneal space. Residual pus may seep continuously into the abdomen, triggering secondary peritonitis or abscess recurrence. Edge-to-edge clip reapproximation isolates infected tissue from the peritoneum instantly. Inflammation fades faster, and patients avoid the need for emergency salvage surgery or prolonged conservative monitoring.

Standardized management protocols for endoscopic drainage complicated by microperforations can be summarized from the therapeutic experience of this case. First, intraoperative prompt detection. After drainage completion, multi-angle thorough inspection of the operative field, with special attention paid to mucosa surrounding the incision, allows early identification of concealed tiny perforations. Second, precise titanium clip closure. Clips are applied layer by layer from perforation rims toward the center for edge-to-edge approximation. Concurrent sealing of the appendiceal opening is conducted when necessary to reinforce closure durability. Third, timely abdominal decompression. Percutaneous gas evacuation is administered to patients with massive intraperitoneal gas and marked abdominal distension to relieve intra-abdominal pressure and mitigate toxic manifestations. Fourth, optimized comprehensive postoperative care. Short-term nil per os orders, sufficient broad-spectrum antibiotics for infection control and intravenous fluid resuscitation are implemented. Serial monitoring of abdominal signs and inflammatory biomarkers is performed, and early enteral feeding is resumed once the patient stabilizes to facilitate accelerated recovery.

This case further validates that early detection paired with primary titanium clip closure and standardized postoperative care can effectively avoid surgical conversion for controllable intraoperative microperforations less than 5 mm in diameter. Its management principle aligns with established strategies for perforations arising from ESD, EFR and other endoscopic procedures. Compared with traditional surgical repair, primary endoscopic closure delivers less trauma, faster postoperative recovery, shorter hospital stays and lower medical costs. It exhibits outstanding clinical advantages, particularly for young patients with intact baseline physical status and well-controlled infection.

Clinicians weigh mainstream treatment tradeoffs before drafting care plans. Low-grade, asymptomatic small abscesses may receive antibiotic monotherapy alone. Long-term medication is mandatory, and pus recurrence is common in follow-up (14). Image-guided percutaneous drainage only removes superficial pus. It cannot eliminate the primary inflammatory lesion at the appendiceal base, so repeated punctures are often needed for relapses (7, 15). Laparoscopic or open appendectomy eradicates infection entirely, yet it brings severe abdominal trauma, longer hospitalization and permanent surgical scars (16, 17). No single treatment fits all patients with appendiceal root abscess; each option has its own indications and risks.

Strict patient screening must be enforced before endoscopic drainage. Candidates qualify only when abscesses remain fully confined to the appendiceal base, colonoscopy tolerance is adequate, and inflammation stays localized without systemic sepsis. Endoscopic intervention is contraindicated for diffuse peritonitis, extensive full-thickness bowel necrosis, multiple organ dysfunction, giant multiloculated abscess cavities or widespread intraperitoneal infection. Emergency surgical debridement or percutaneous drainage is selected for these high-risk groups. Intraoperative safety steps include slow blunt dissection, repeated cavity irrigation, real-time EUS lesion localization and prompt decompression for accumulated intraperitoneal gas to lower complication odds.

Most endoscopic treatments for appendiceal conditions are limited to uncomplicated acute appendicitis. Few clinical reports address periappendiceal abscess masquerading as submucosal lesions and its endoscopic diagnosis and therapy. This case proves EUS can precisely distinguish such atypical lesions. Endoscopic incision and drainage yields favorable curative effects with controllable adverse events, representing a safe, novel minimally invasive strategy for this rare abscess variant.

## Limitations

This study has several inherent limitations. Data were collected from only one patient at a single center without matched controls, so the therapeutic outcomes cannot be generalized to all patients with appendiceal root abscess. The two-month follow-up period is insufficient to evaluate long-term abscess recurrence and tissue recovery, and prolonged long-term monitoring is necessary to obtain solid prognostic evidence. Moreover, we did not perform comparative analyses against laparoscopic/open appendectomy or percutaneous drainage. Existing data fail to verify the superiority of this endoscopic strategy, and large multicenter prospective controlled trials are required to objectively compare the safety and efficacy of different treatment modalities.

## Conclusions

Periappendiceal abscess presenting as submucosal mass at the cecal appendiceal root carries insidious clinical manifestations and is susceptible to misdiagnosis. Endoscopic ultrasonography acts as the core tool to establish definitive diagnosis. Endoscopic incision and drainage is a safe, minimally invasive and efficacious intervention. Intraoperative minor perforations can be successfully managed through primary titanium clip closure combined with conservative regimens, eliminating the need for laparotomy. This therapeutic model stands as a preferred strategy for this atypical periappendiceal abscess subtype and merits widespread clinical promotion.

## Data Availability

The original contributions presented in the study are included in the article/Supplementary Material, further inquiries can be directed to the corresponding author.

## Supplementary material

Supplementary Figure S1 Follow-up abdominal CT findings after treatment.
